# Caloric canal paresis thresholds in relation to vHIT-defined acute unilateral vestibular dysfunction: a retrospective analysis

**DOI:** 10.3389/fneur.2026.1893212

**Published:** 2026-09-14

**Authors:** Jae Woo Lee, Mingyeong Kim, Gi-Sung Nam, Hyun Su Lee, Young Joon Seo, Tae Hoon Kong

**Affiliations:** 1 Department of Otorhinolaryngology – Head and Neck Surgery, Yonsei University Wonju College of Medicine, Wonju, Republic of Korea; 2 Department of Bio-AI Convergence, Yonsei University Graduate School, Wonju, Republic of Korea; 3 Laboratory of Otology Neurology Artificial Intelligence Research (ONAIR), Yonsei University Wonju College of Medicine, Wonju, Republic of Korea; 4 Department of Otorhinolaryngology – Head and Neck Surgery, Chosun University College of Medicine, Gwangju, Republic of Korea; 5 Research Institute of Hearing Enhancement, Yonsei University Wonju College of Medicine, Wonju, Republic of Korea

**Keywords:** acute unilateral vestibulopathy, caloric test, canal paresis threshold, vestibular neuritis, video head impulse test

## Abstract

**Background:**

Caloric testing and the video head impulse test (vHIT) assess vestibular function at different frequency ranges and provide complementary information in patients with acute vertigo. Although a canal paresis (CP) threshold of 25% is commonly used to define caloric abnormality, interpreting CP values in relation to vHIT-defined unilateral high-frequency vestibular dysfunction remains clinically challenging.

**Objective:**

We evaluated how conventional and alternative caloric CP thresholds align with vHIT-defined acute unilateral vestibular dysfunction and sought an interpretive CP threshold best corresponding to vHIT-defined findings.

**Methods:**

This retrospective analysis included patients presenting with acute vertigo within 72 h of symptom onset who underwent both caloric testing and vHIT at a tertiary referral center. Patients with clinically suspected benign paroxysmal positional vertigo, Ménière’s disease, vestibular migraine, sudden sensorineural hearing loss or labyrinthitis with acute auditory symptoms, central vestibular disorders, bilateral vestibulopathy, or bilateral caloric weakness were excluded. vHIT-defined unilateral dysfunction was based on compatible clinical features and abnormal horizontal-canal vHIT findings indicating unilateral high-frequency vestibulo-ocular reflex dysfunction. Receiver operating characteristic (ROC) analysis examined how caloric CP values corresponded to this classification.

**Results:**

Of 1,634 eligible patients, 145 met criteria for vHIT-defined unilateral dysfunction. The symptomatic vHIT-normal comparison group comprised 1,489 patients without a final diagnosis of definite peripheral or central vestibular disease, although some had isolated caloric abnormalities. The conventional 25% CP threshold showed high sensitivity for the vHIT-based classification but classified a larger number of symptomatic vHIT-normal patients as CP abnormal. ROC analysis identified an approximately 30% CP threshold (Youden’s index) with sensitivity 0.945 and specificity 0.867. This threshold reduced vHIT-discordant caloric abnormal classifications compared with 25% while maintaining close correspondence with the vHIT-defined classification.

**Conclusion:**

In patients evaluated within 72 h of acute vertigo onset, a CP threshold of approximately 30% showed the closest correspondence with vHIT-defined unilateral high-frequency vestibular dysfunction. It should be interpreted as a practical benchmark for interpreting caloric CP in relation to vHIT findings, not as an independent diagnostic criterion for AUVP.

## 1 Introduction

Acute unilateral vestibulopathy (AUVP), also known as vestibular neuritis, is a common peripheral vestibular disorder with an estimated annual incidence of approximately 3.5–10.5 per 100,000 individuals (1, 2). It most commonly affects adults between 30 and 60 years of age and represents the third most frequent peripheral vestibulopathy, following benign paroxysmal positional vertigo and Ménière’s disease (1, 3). Although viral infection and ischemic mechanisms involving the vestibular artery have been proposed, the exact pathophysiology of AUVP remains incompletely understood (4, 5).

The Bárány Society published updated diagnostic criteria for AUVP in 2022, emphasizing both characteristic clinical features and objective evidence of vestibular dysfunction (3). The criteria define AUVP as the acute or subacute onset of sustained vertigo lasting at least 24 h, accompanied by spontaneous peripheral vestibular nystagmus, and require “unambiguous evidence” of reduced vestibulo-ocular reflex (VOR) function on the side opposite to the fast phase of the spontaneous nystagmus. This unambiguous evidence refers to a quantitatively demonstrable deficit identified using objective vestibular tests, most commonly the caloric test and the video head impulse test (vHIT) (3).

The caloric test, introduced by Robert Bárány in 1906, has long been regarded as the gold standard for detecting vestibular hypofunction (6). It evaluates vestibular function at an extremely low, non-physiological frequency (approximately 0.003 Hz) and commonly quantifies asymmetry using canal paresis (CP) calculated with Jongkees’ formula, with a conventional pathological threshold of 25% (7, 8). However, caloric testing shows substantial inter-individual variability and may fail to demonstrate vestibular dysfunction in certain pathological conditions, particularly during the acute phase of AUVP (5, 9). Indeed, the relative diagnostic roles of caloric testing and vHIT in acute vertigo have been increasingly debated, with some authors questioning whether caloric testing remains necessary when vHIT is available (10, 11).

In contrast, the vHIT assesses VOR function at physiologically relevant high frequencies (1–5 Hz) and has demonstrated clinical utility for detecting high-frequency VOR deficits in patients with AUVP (12–14). Reduced VOR gain (<0.7) or significant interaural gain asymmetry (>0.3), particularly when accompanied by corrective saccades, is widely accepted as objective evidence of unilateral high-frequency vestibular dysfunction. In clinical practice, dissociation between caloric test results and vHIT findings is frequently observed, including cases with abnormal caloric responses despite normal vHIT findings and cases with vHIT abnormalities despite preserved caloric responses (9, 13, 15).

This dissociation raises an important interpretive question: when unilateral high-frequency VOR dysfunction is identified by vHIT in patients with acute vertigo, how should caloric asymmetry be interpreted in relation to conventional CP thresholds? Importantly, although vHIT has demonstrated clinical utility for detecting high-frequency VOR deficits in AUVP, it should not be regarded as an independent gold standard for AUVP, because AUVP is a clinical diagnosis that requires integration of symptoms, bedside findings, auditory features, central signs, and objective vestibular testing (3, 12–14). Because the Bárány Society criteria accept either an impaired unilateral vHIT gain or a unilateral reduced caloric response as the required “unambiguous evidence” of a VOR deficit, the two tests are effectively treated as alternative means of satisfying the same diagnostic requirement, even though they probe different frequency ranges. The conventional 25% CP threshold, however, was derived from laboratory-specific normative data rather than from studies validating it against this diagnostic use, and its appropriateness in the acute phase of AUVP has not been systematically examined (8, 9).

There is therefore little empirical guidance on what caloric result is expected when a VOR deficit is defined by vHIT, or on whether 25% is the appropriate CP threshold for this purpose. Characterizing how caloric CP values correspond to vHIT-defined unilateral dysfunction would provide a practical, data-driven aid for interpreting caloric findings within this diagnostic framework. Accordingly, we aimed to examine how conventional and alternative caloric CP thresholds align with vHIT-defined unilateral high-frequency vestibular dysfunction and to provide practical evidence for interpreting caloric findings within a multimodal vestibular assessment framework.

## 2 Materials and methods

### 2.1 Participants

We conducted a retrospective study at a single tertiary university hospital. A total of 2,788 patients who visited our hospital for acute vertigo and underwent both caloric testing and vHIT within 72 h of symptom onset between January 2016 and December 2023 were screened (Figure 1). Among these patients, individuals younger than 18 years (*n* = 119) or older than 70 years (*n* = 636) were excluded. Patients were also excluded if they could not complete either caloric testing or vHIT, if either test was technically inadequate, or if a central vestibular cause was suspected or confirmed (*n* = 399). Technically inadequate tests included incomplete caloric irrigation, missing or unreliable eye movement recordings, calibration failure, excessive artifacts, insufficient valid head impulses, goggle slippage, or head impulses outside the acceptable velocity and acceleration ranges.

**Figure 1:**
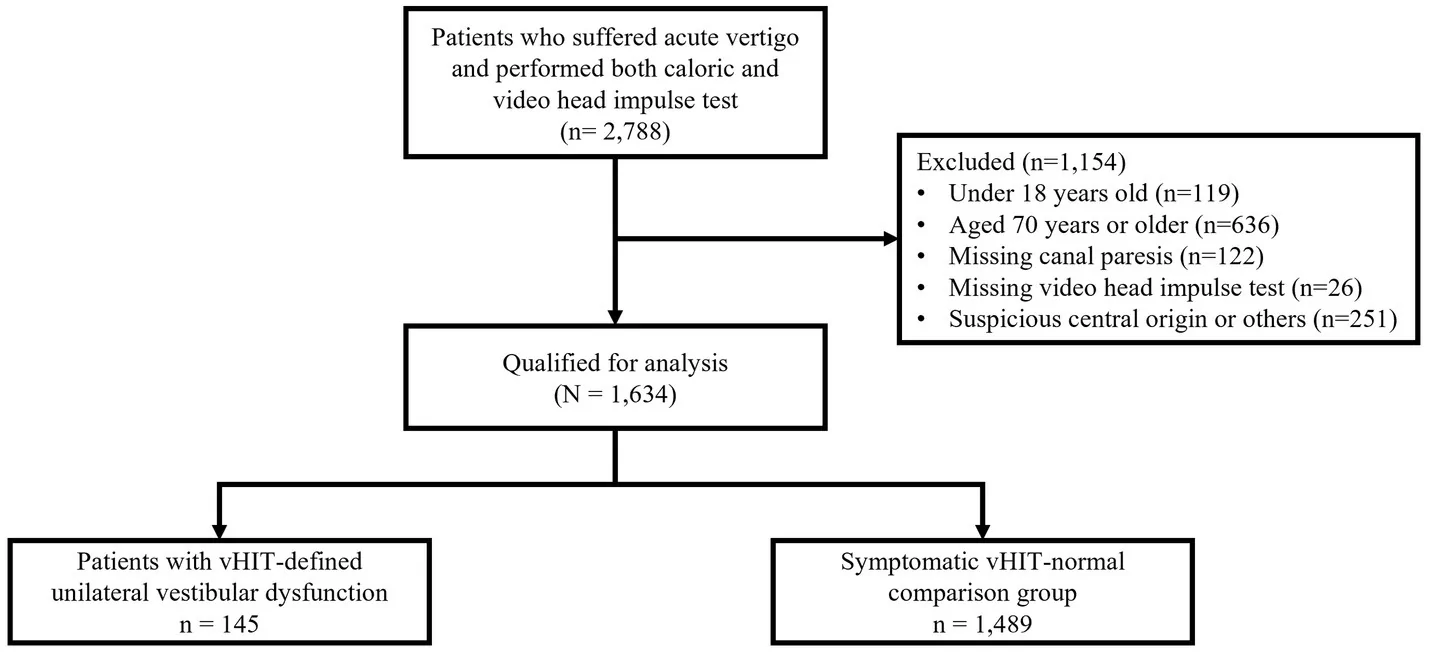
Flow diagram illustrating patient selection and the study design. A total of 2,788 patients presenting with acute vertigo underwent both caloric testing and video head impulse testing (vHIT). After exclusion based on age, incomplete examinations, and suspected central causes, 1,634 patients were included in the final analysis and classified into the vHIT-defined unilateral vestibular dysfunction and symptomatic vHIT-normal comparison groups.

Patients were diagnosed with AUVP based on a review of medical records, following the diagnostic criteria proposed by the Bárány Society: (1) acute or subacute onset of continuous vertigo lasting at least 24 h; (2) spontaneous, direction-fixed horizontal nystagmus enhanced by removal of visual fixation; (3) decreased VOR function on the side opposite to the fast phase of the nystagmus; and (4) absence of central nervous system disorders resulting in vertigo and nystagmus.

Patients with acute auditory symptoms suggestive of labyrinthitis, sudden sensorineural hearing loss with vertigo, or Ménière’s disease were not classified as AUVP. Patients with clinically suspected benign paroxysmal positional vertigo based on positional testing, Ménière’s disease based on recurrent vertigo with fluctuating auditory symptoms, vestibular migraine according to Bárány Society/International Headache Society criteria, central vestibular disorders based on ocular motor findings or neuroimaging, bilateral vestibulopathy, or bilateral caloric weakness were excluded from the vHIT-normal comparison group. Thus, the symptomatic vHIT-normal comparison group consisted of patients who presented with transient dizziness/vertigo or acute dizziness and did not receive a final diagnosis of definite peripheral or central vestibular disease after neurotologic evaluation. Because some patients in this group showed isolated caloric abnormalities despite normal vHIT findings, this group should not be interpreted as a physiologically normal control group.

In this study, vHIT-defined unilateral vestibular dysfunction was based primarily on horizontal-canal findings. Abnormal horizontal-canal vHIT was defined by reduced VOR gain on the clinically affected side (<0.7), significant interaural gain asymmetry (>0.3), and reproducible corrective catch-up saccades concordant with the clinically affected side, consistent with previous studies using head impulse testing or vHIT to identify unilateral vestibular hypofunction (12–14, 16). Corrective saccades were considered supportive evidence of unilateral vestibular hypofunction when repeatedly observed after head impulses toward the affected side. Vertical-canal vHIT findings were reviewed when available to characterize the pattern of vestibular involvement, but they were not used as the primary classification criterion in the present caloric-vHIT analysis because caloric testing primarily assesses horizontal-canal function.

Neurotologic examinations—including assessments of spontaneous and gaze-evoked nystagmus, smooth pursuit, saccadic eye movements, limb ataxia, and balance function—were performed in all patients. Patients presenting with isolated vertigo who exhibited central ocular motor signs, limb ataxia, or severe postural imbalance underwent brain magnetic resonance imaging; those with confirmed central lesions were excluded from the study.

Final clinical impressions were adjudicated by one experienced otologist/vestibular specialist (T.H.K.) based on the full clinical course and neurotologic evaluation. The review incorporated clinical history, vertigo duration and pattern, bedside neurotologic examination, spontaneous and gaze-evoked nystagmus, positional test results, auditory symptoms, vestibular function test results, and neuroimaging findings when available. The adjudicator was blinded to the study-specific caloric CP threshold classifications of 25, 30, and 34% and to the results of the threshold-based analysis. Because vHIT and caloric testing were performed as part of routine clinical care, complete blinding to all vestibular test information was not feasible. This study was approved by the Institutional Review Board of our institution, which waived the requirement for informed consent (Approval No. CR323027).

### 2.2 Assessment of vestibular function

Caloric tests were performed using the Interacoustics VisualEyes™ system and the Aqua Stim™ irrigator (Middelfart, Denmark). Water irrigations were conducted using both cold (30 °C) and warm (44 °C) water for 30 s. A total volume of 250 mL was administered while patients were in the supine position with 30° head flexion to optimize canal stimulation. The irrigations were performed in the following sequence: cold water in the right ear, cold water in the left ear, warm water in the right ear, and warm water in the left ear (cold/cold/warm/warm). Intervals of 200 s were maintained between irrigations to allow for recovery. The convection flow induced in the inner ear fluid elicited horizontal eye movements, which were recorded in darkness using a calibrated video-oculography device. The mean peak slow-phase velocity was measured over a 10-s period to assess vestibular function.

Canal paresis was calculated using Jongkees’ formula, as previously described (7, 8): CP (%) = [(right warm + right cold) − (left warm + left cold)] / (right warm + right cold + left warm + left cold) × 100. The absolute CP value was used for threshold analysis, and the side of weakness was determined according to the direction of asymmetry. Bilateral caloric weakness was defined according to our laboratory criterion as a total bithermal peak slow-phase velocity of <20°/s across the four irrigations. Patients with bilateral caloric weakness were excluded because CP based on relative asymmetry may be difficult to interpret in the presence of bilateral hypofunction. According to the laboratory protocol, patients were instructed to withhold vestibular suppressants, sedatives, antihistamines, benzodiazepines, and antiemetic medications for 48 h before vestibular testing whenever clinically feasible. In the acute-care setting, if complete medication washout could not be ensured, testing was performed after the medication effect was considered clinically minimized. Caloric and vHIT systems underwent routine calibration and preventive maintenance according to the manufacturer’s recommendations, and device calibration was checked before testing.

The vHIT was performed using the Interacoustics EyeSeeCam™ system. The test involved rapid head impulses with a peak head velocity ranging from 100°/s to 250°/s and a peak head acceleration between 1,000°/s^2^ and 2,500°/s^2^. The test was conducted for the lateral, anterior, and posterior semicircular canals. Each head impulse was repeated 10–15 times, with a minimum of 10 successful impulses per canal to ensure reliable data. The vHIT assessed VOR gain and the presence of compensatory CUS.

### 2.3 Statistical analysis

Continuous variables between the vHIT-defined unilateral vestibular dysfunction group and the symptomatic vHIT-normal comparison group were compared using the Student’s *t*-test for unpaired groups. Categorical variables were compared using the chi-square test or Fisher’s exact test, as appropriate.

The primary analysis evaluated how caloric CP thresholds corresponded to vHIT-defined unilateral high-frequency vestibular dysfunction. ROC curve analysis was performed to examine how caloric CP values aligned with vHIT-defined classification. Youden’s index was calculated to identify the CP threshold that maximized the combined sensitivity and specificity relative to the vHIT-based classification. Youden’s index was defined as J = sensitivity + specificity – 1 (17). The Youden’s index-derived threshold was compared with the conventional 25% threshold and with the sensitivity-specificity intersection threshold.

CP-abnormal/vHIT-normal cases were interpreted as vHIT-discordant caloric abnormalities, as vHIT cannot be considered an independent disease reference standard. Sensitivity, specificity, and the area under the ROC curve were calculated to quantify how well different caloric CP thresholds corresponded to the predefined vHIT-based classification. These measures were used to evaluate alignment with the reference classification rather than to establish the independent diagnostic accuracy of caloric testing for AUVP. Ninety-five percent confidence intervals were calculated for sensitivity, specificity, and AUC. The area under the ROC curve was reported to describe the overall correspondence between caloric CP values and vHIT-defined classification. All statistical analyses were performed using Python version 3.11.7 (Python Software Foundation, Wilmington, DE, United States). A *p*-value of less than 0.05 was considered statistically significant.

## 3 Results

Among the 1,634 patients eligible for analysis, 145 (8.9%) met the criteria for vHIT-defined unilateral vestibular dysfunction in the clinical context of AUVP, and the remaining 1,489 (91.1%) were assigned to the symptomatic vHIT-normal comparison group. Patients in the comparison group demonstrated normal horizontal-canal vHIT findings—defined as VOR gain ≥0.7 on both sides, interaural gain asymmetry <0.3, and absence of reproducible corrective catch-up saccades. This group was not intended to represent healthy controls. Rather, it consisted of patients with acute dizziness/vertigo in whom definite peripheral or central vestibular disorders were excluded after clinical evaluation.

An overview of the clinical characteristics of the patients, comparing those with AUVP and the symptomatic vHIT-normal comparison group, is provided in Table 1. The mean age of the overall cohort was 51.6 ± 12.8 years. Patients with AUVP were slightly older, with a mean age of 53.8 ± 11.4 years, compared with the symptomatic vHIT-normal comparison group, which had a mean age of 51.4 ± 12.9 years. This age difference between the two groups was statistically significant (*p =* 0.033). The total study population consisted of 605 males and 1,029 females. A significant difference in sex distribution was observed: the AUVP group showed a male-to-female ratio of approximately 1.54:1 (88 males vs. 57 females), whereas the symptomatic vHIT-normal comparison group exhibited a reversed ratio of approximately 1:1.88 (517 males vs. 972 females). The difference in sex distribution between the groups was highly significant (*p <* 0.001). Among patients with AUVP, the affected side was nearly evenly distributed, with 79 cases on the right and 66 cases on the left. Patients with AUVP demonstrated a significantly higher degree of CP (mean = 69.4 ± 23.0%) compared with the symptomatic vHIT-normal comparison group (mean = 15.1 ± 15.7%) and the overall population (mean = 19.9 ± 22.6%), with a *p*-value of < 0.001. Similarly, the mean directional preponderance was higher in patients with AUVP (21.2 ± 18.0%) compared with the symptomatic vHIT-normal comparison group (10.9 ± 8.8%) and the total population (11.8 ± 10.3%). This difference was also statistically significant (*p <* 0.001). In the vHIT analysis, patients with AUVP demonstrated a mean gain of 0.39 ± 0.16 on the lesion side and 0.88 ± 0.12 on the unaffected side. For the symptomatic vHIT-normal comparison group, lacking a lesion side, gains for the right and left sides were analyzed separately, resulting in mean values of 0.97 ± 0.13 and 0.98 ± 0.13, respectively. Corrective catch-up saccades consistent with the clinically affected side were observed in all patients classified as having vHIT-defined unilateral vestibular dysfunction, whereas no consistent corrective saccades were identified in the symptomatic vHIT-normal comparison group.

**Table 1:** Clinical and demographic characteristics of patients with vHIT-defined unilateral vestibular dysfunction and symptomatic patients with normal vHIT findings.

Variable	Total (*n* = 1,634)	vHIT-defined unilateral vestibular dysfunction (*n* = 145)	Symptomatic vHIT-normal group (*n* = 1,489)	*p*-value
Age (years, mean±SD)	51.6 ± 12.8	53.8 ± 11.4	51.4 ± 12.9	0.033^*^
Sex (Male:Female)	605:1029	88:57	517:972	*p <* 0.001
Affected side (Rt:Lt)	79:66	79:66	NA	Not tested
Caloric test				
Canal paresis (%, mean±SD)	19.9 ± 22.6	69.4 ± 23.0	15.1 ± 15.7	*p <* 0.001
Directional preponderance (%, mean±SD)	11.8 ± 10.3	21.2 ± 18.0	10.9 ± 8.8	*p <* 0.001
Video head impulse test				
Affected-side/right-side vHIT gain (mean ± SD)		0.39 ± 0.16	0.97 ± 0.13†	*p <* 0.001
Unaffected-side/left-side vHIT gain (mean ± SD)		0.88 ± 0.12	0.98 ± 0.13‡	*p <* 0.001
CUS rate (%)	145 (8.87%)	145 (100%)	0 (0%)	not tested

For vHIT gain values, affected-side and unaffected-side values refer to the clinically affected and unaffected sides in the vHIT-defined unilateral vestibular dysfunction group. In the symptomatic vHIT-normal comparison group, right- and left-side horizontal-canal gain values are shown because no lesion side was assigned. Values are presented as mean ± standard deviation for continuous variables, and as numbers with percentages for categorical variables. CUS rate was not statistically compared because corrective saccades were part of the vHIT-based clinical classification. * *p* < 0.05, ^†^Rt, ^‡^Lt.

Final clinical adjudication of the symptomatic vHIT-normal comparison group showed that approximately 1,191 patients (80.0%) did not receive a final diagnosis of definite peripheral or central vestibular disease after neurotologic evaluation and were judged to have clinically transient or non-definite vestibular symptoms. Approximately 74 patients (5.0%) were classified as having transient vestibular attack or transient dizziness. The remaining 224 patients (15.0%) had non-vestibular or nonspecific causes of dizziness, including cardiogenic dizziness and nonspecific dizziness. Importantly, because some patients in the vHIT-normal comparison group had isolated caloric abnormalities, this group should not be considered a physiologically normal control group.

### 3.1 Youden’s index-derived CP threshold in relation to vHIT-defined dysfunction

The Youden’s index-derived CP threshold that best corresponded to vHIT-defined unilateral vestibular dysfunction was 30% (Figure 2). At this threshold, Youden’s index was 0.81, and the area under the ROC curve was 0.950 (95% CI, 0.925–0.975), indicating strong alignment between caloric CP values and vHIT-defined classification. Because vHIT was not used as an independent disease reference standard, this result should be interpreted as correspondence with vHIT-defined high-frequency vestibular dysfunction rather than independent diagnostic discrimination for AUVP.

**Figure 2:**
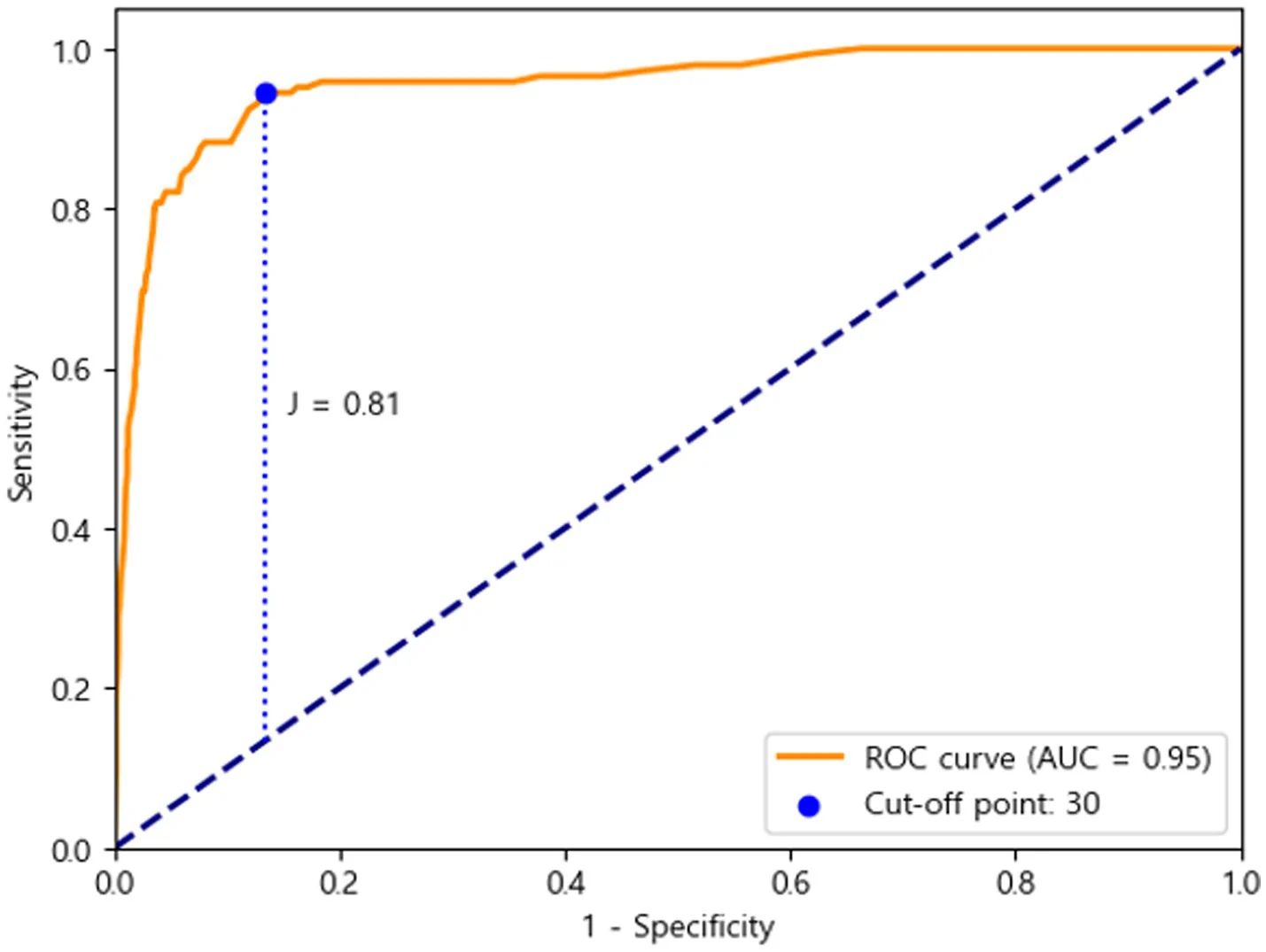
Receiver operating characteristic (ROC) curve analysis of caloric canal paresis (CP) in relation to vHIT-defined unilateral vestibular dysfunction. The CP threshold derived from Youden’s J statistic is shown as the point maximizing the combined sensitivity and specificity for alignment with vHIT-defined classification. The area under the ROC curve, 0.950 (95% CI, 0.925–0.975), illustrates the overall correspondence of CP values with vHIT-defined unilateral high-frequency vestibular dysfunction, rather than independent diagnostic accuracy for AUVP.

### 3.2 Comparative analysis of CP thresholds in relation to vHIT-defined dysfunction

To compare the interpretive performance of different CP thresholds, the conventional threshold of 25%, the Youden’s index-derived threshold of 30%, and the sensitivity-specificity intersection threshold of 34% were evaluated (Figure 3). The conventional 25% threshold showed high sensitivity for the vHIT-based classification (0.959), but it also classified a larger number of symptomatic vHIT-normal patients as CP abnormal. The 30% threshold showed sensitivity of 0.945 and specificity of 0.867 relative to the vHIT-based classification, indicating improved specificity with only a small reduction in sensitivity. The 34% threshold further increased specificity to 0.897 but was associated with a greater reduction in sensitivity to 0.883.

**Figure 3:**
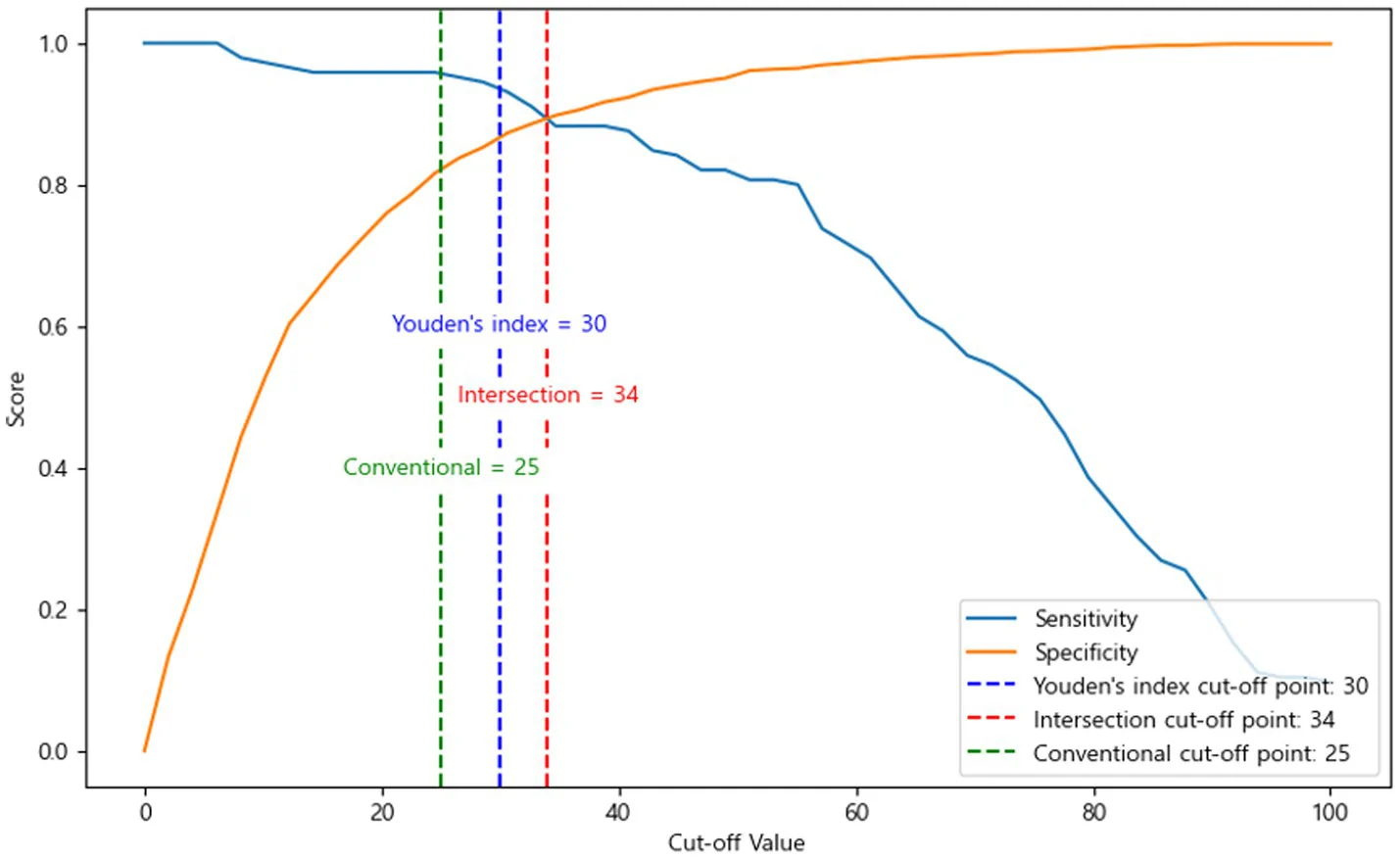
Comparison of sensitivity and specificity across caloric canal paresis (CP) thresholds in relation to vHIT-defined unilateral vestibular dysfunction. The conventional threshold of 25%, the Youden’s index-derived threshold of 30%, and the sensitivity-specificity intersection threshold of 34% are shown.

Because vHIT served as a reference classification rather than a disease gold standard, and because caloric (~0.003 Hz) and vHIT (1–5 Hz) assess different frequency ranges, these sensitivity and specificity values describe how closely caloric CP thresholds corresponded to the vHIT-based classification rather than diagnostic accuracy for AUVP; the residual discordant cases are consistent with genuine frequency-specific dissociation rather than test error.

### 3.3 Corresponding and discordant classifications at different CP thresholds

The classification outcomes for these thresholds are presented in Table 2. At the conventional CP threshold of 25%, 1,215 patients were classified as CP-normal/vHIT-normal and 139 as CP-abnormal/vHIT-defined unilateral vestibular dysfunction, whereas 274 symptomatic vHIT-normal patients were classified as CP abnormal and 6 patients with vHIT-defined unilateral vestibular dysfunction were classified as CP normal. At the Youden’s index-derived threshold of 30%, the number of CP-abnormal/vHIT-normal discordant cases decreased from 274 to 198, while CP-normal/vHIT-defined unilateral vestibular dysfunction cases increased slightly from 6 to 8. At the intersection threshold of 34%, CP-abnormal/vHIT-normal discordant cases further decreased to 154, but CP-normal/vHIT-defined unilateral vestibular dysfunction cases increased to 17. These findings indicate that the 30% threshold provided a clinically reasonable balance between reducing vHIT-discordant caloric abnormalities and maintaining correspondence with vHIT-defined unilateral vestibular dysfunction.

**Table 2:** Classification correspondence between caloric canal paresis thresholds and vHIT-defined unilateral vestibular dysfunction.

vHIT classification	CP normal	CP abnormal	Sensitivity	Specificity
Cut-off value: 25%				
Symptomatic vHIT-normal group	1,215	274	0.959 (95% CI, 0.913–0.981)	0.816 (95% CI, 0.795–0.835)
vHIT-defined unilateral vestibular dysfunction	6	139		
Cut-off value: 30%				
Symptomatic vHIT-normal group	1,291	198	0.945 (95% CI, 0.895–0.972)	0.867 (95% CI, 0.849–0.883)
vHIT-defined unilateral vestibular dysfunction	8	137		
Cut-off value: 34%				
Symptomatic vHIT-normal group	1,335	154	0.883 (95% CI, 0.820–0.925)	0.897 (95% CI, 0.880–0.911)
vHIT-defined unilateral vestibular dysfunction	17	128		

Classification outcomes are shown for the conventional CP threshold of 25%, the Youden’s index-derived threshold of 30%, and the sensitivity-specificity intersection threshold of 34%, using vHIT-defined unilateral vestibular dysfunction as the reference classification. CP-abnormal/vHIT-normal cases and CP-normal/vHIT-defined unilateral vestibular dysfunction cases indicate discordant classifications and should not be interpreted as definitive diagnostic misclassifications for AUVP because vHIT was not used as an independent disease reference standard. AUVP, acute unilateral vestibulopathy; CP, canal paresis; vHIT, video head impulse test; CI, confidence interval.

## 4 Discussion

The present study evaluated how caloric CP thresholds corresponded to vHIT-defined unilateral high-frequency vestibular dysfunction in patients presenting with acute vertigo within 72 h of symptom onset. The conventional 25% CP threshold showed high sensitivity for alignment with vHIT-defined unilateral vestibular dysfunction, but it also classified a larger number of symptomatic vHIT-normal patients as CP abnormal. A CP threshold of approximately 30% improved specificity relative to the vHIT-based classification from 0.816 to 0.867 while maintaining high sensitivity at 0.945. This shift reduced CP-abnormal/vHIT-normal discordant classifications from 274 to 198 cases, with only a small increase in CP-normal/vHIT-defined unilateral vestibular dysfunction cases. Importantly, these findings should not be interpreted as establishing an independent diagnostic CP threshold for AUVP. Rather, the 30% threshold should be understood as an interpretive benchmark for caloric CP in relation to vHIT-defined findings within a multimodal vestibular assessment framework.

### 4.1 Interpretation of caloric testing in relation to vHIT-defined unilateral vestibular dysfunction

The caloric test has long been regarded as the gold standard for assessing unilateral vestibular hypofunction since its introduction by Bárány and its subsequent quantification using the Jongkees formula (6, 7). The commonly used CP cut-off of 20–25% was historically derived from laboratory-specific norms rather than outcome-based validation studies (8). Consequently, the appropriateness of a rigid 25% threshold has been questioned in previous clinical investigations (5, 9).

Several studies have reported that patients with clinically definite vestibular neuritis may exhibit CP values below 25%, a phenomenon often referred to as “minimal CP,” particularly in cases with milder symptoms or rapid recovery (9). Conversely, studies of caloric-vHIT dissociation in dizzy patients have shown that abnormal caloric responses can have different clinical implications depending on lesion location and etiology (13). Our findings are consistent with these observations and suggest that modestly higher CP thresholds may improve correspondence with vHIT-defined unilateral high-frequency vestibular dysfunction. However, CP-abnormal/vHIT-normal cases should not automatically be interpreted as diagnostic misclassifications, because caloric testing may detect genuine low-frequency vestibular dysfunction in some clinical contexts. For this reason, these findings are best understood as caloric-vHIT discordance rather than as evidence of diagnostic error.

### 4.2 Pathophysiological rationale: frequency-specific vestibular assessment

The observed discrepancy between caloric and vHIT findings is largely attributable to the different frequency domains assessed by the two tests. Caloric stimulation evaluates vestibular function at an extremely low, non-physiological frequency of approximately 0.003 Hz, whereas vHIT assesses high-frequency, physiologically relevant VOR function during rapid head movements, typically within the range of 1–5 Hz (8, 12, 16). Because these tests probe different aspects of semicircular canal function, discordant results should be interpreted as reflecting frequency-dependent vestibular impairment rather than simple test inconsistency.

This frequency-specific dissociation has been reported in approximately 18–36% of patients presenting with dizziness (13, 14). Comparable dissociation between vHIT and caloric findings has been documented directly in patients evaluated during acute vertigo and across sub-acute and chronic vestibular disorders (11, 18). The most common pattern is abnormal caloric responses with normal vHIT findings, particularly in peripheral vestibular disorders such as vestibular neuritis and Ménière’s disease (13, 19). Conversely, abnormal vHIT findings with relatively preserved caloric responses may occur when high-frequency VOR dysfunction predominates (20). These observations support the need to interpret caloric CP values in relation to the high-frequency vestibular deficits identified by vHIT.

In AUVP, acute impairment of unilateral high-frequency VOR function is a core physiological feature, making vHIT a clinically relevant reference for identifying the affected vestibular side (15, 20). In this context, the present study evaluated how low-frequency vestibular asymmetry measured by caloric testing aligns with vHIT-defined unilateral high-frequency vestibular dysfunction. The identified CP threshold should therefore be understood as a frequency-domain–specific refinement of caloric interpretation in vHIT-defined unilateral vestibular dysfunction, rather than as evidence that caloric testing and vHIT should yield identical classifications in all patients.

Although vHIT has been reported to be clinically useful for identifying high-frequency VOR deficits in AUVP, its diagnostic performance depends on disease stage, affected vestibular division, technical quality, and the criteria used for defining abnormal gain and corrective saccades (12–14, 16, 20). Therefore, vHIT should be interpreted as one component of objective vestibular assessment rather than as a standalone diagnostic gold standard. This consideration was central to the framing of the present study, in which vHIT was used as an objective reference classification for unilateral high-frequency vestibular dysfunction rather than as the diagnostic gold standard for AUVP.

### 4.3 Clinical meaning of the symptomatic vHIT-normal comparison group

A key methodological consideration is the interpretation of the symptomatic vHIT-normal comparison group. This group was not composed of asymptomatic healthy controls and should not be interpreted as a physiologically normal population. However, it was also not intended to represent a heterogeneous group of established peripheral vestibular disorders. Patients with clinically suspected BPPV, Ménière’s disease, vestibular migraine, sudden sensorineural hearing loss or labyrinthitis with acute auditory symptoms, central vestibular disorders, bilateral vestibulopathy, or bilateral caloric weakness were excluded. Clinical adjudication showed that most patients in this group did not receive a final diagnosis of definite peripheral or central vestibular disease after neurotologic evaluation, whereas a smaller proportion had transient vestibular attack/transient dizziness or non-vestibular/nonspecific dizziness.

Nevertheless, some patients in the vHIT-normal comparison group had isolated caloric abnormalities. Such findings should not be dismissed as false-positive caloric results, because dissociation between pathological caloric responses and normal vHIT findings may reflect genuine low-frequency vestibular dysfunction and has been reported in several vestibular disorders, including Ménière’s disease and vestibular migraine (21, 22). Therefore, CP-abnormal/vHIT-normal cases in the present study were interpreted as vHIT-discordant caloric abnormalities rather than diagnostic misclassifications. The present results should be interpreted specifically within the context of acute vertigo patients in whom definite peripheral and central vestibular disorders were excluded through clinical evaluation.

### 4.4 Clinical implications in the context of Bárány Society criteria

The 2022 Bárány Society diagnostic criteria for AUVP require “unambiguous evidence” of unilateral VOR dysfunction but deliberately avoid specifying numerical cut-offs for either caloric testing or vHIT due to a lack of universal consensus (3). Although this approach accommodates methodological variability, it also introduces uncertainty in clinical interpretation.

Our findings provide empirical data that may help refine the interpretation of caloric test results within this framework. Specifically, when vHIT demonstrates clear unilateral high-frequency VOR impairment in the acute phase, a caloric CP value of approximately 30% may provide more consistent supportive evidence of unilateral vestibular hypofunction than the traditional 25% threshold. Conversely, CP values between 25 and 30% should not be dismissed but may require more cautious interpretation in relation to clinical findings, vHIT results, auditory symptoms, and other vestibular test outcomes.

From a practical standpoint, when caloric testing is used to provide the “unambiguous evidence” of a VOR deficit required by the Bárány criteria, interpreting the result against a modestly higher benchmark of approximately 30% rather than the conventional 25% has two practical implications in the acute phase. First, it reduces the number of symptomatic vHIT-normal patients classified as CP-abnormal from 274 to 198 (a 28% reduction), limiting the over-interpretation of isolated low-frequency caloric asymmetry as evidence of AUVP, while reclassifying only two additional vHIT-defined cases as CP-normal. Second, because the correspondence between the two tests is only partial, a caloric–vHIT disagreement should not be dismissed: an abnormal caloric with a normal vHIT may reflect genuine isolated low-frequency dysfunction. The two tests are therefore best used complementarily within a multimodal assessment rather than as freely interchangeable evidence, and the 30% value should be regarded as an interpretive aid, not a stand-alone diagnostic criterion.

### 4.5 Complementary roles of vHIT and caloric testing

The present findings do not support replacing caloric testing with vHIT. Instead, they underscore the complementary roles of these two modalities. Although some authors have argued that the routine role of caloric testing should be reconsidered in the vHIT era (10), our findings support a complementary rather than a substitutive relationship between the two tests. vHIT serves as a rapid and physiologically relevant screening tool in the acute setting, whereas caloric testing provides valuable information regarding low-frequency vestibular function and long-term deficits (8, 15). Caloric testing remains particularly useful for detecting isolated low-frequency dysfunction, monitoring chronic or fluctuating vestibular disorders such as Ménière’s disease, and documenting residual vestibular weakness during follow-up when vHIT results are equivocal or technically limited (20, 23, 24).

In selected patients with vHIT-defined unilateral vestibular dysfunction, interpreting caloric results with awareness of a modestly higher CP threshold may enhance interpretive confidence by reducing vHIT-discordant caloric abnormalities among symptomatic vHIT-normal patients, while preserving the complementary value of caloric testing.

These findings are consistent with previous recommendations that vHIT and caloric testing should be used complementarily rather than interchangeably (8, 15, 25). vHIT provides rapid information regarding high-frequency VOR function and may be useful in acute clinical workflows, whereas caloric testing remains important for assessing low-frequency vestibular function and identifying caloric-vHIT dissociation. The present study does not argue for replacing caloric testing with vHIT or for excluding caloric testing from the evaluation of acute vertigo. Instead, it provides an interpretive framework for understanding CP thresholds when both tests are available.

### 4.6 Limitations

This study should be interpreted considering several limitations. First, vHIT-defined unilateral vestibular dysfunction was used as the reference classification for evaluating caloric CP thresholds. Therefore, the present analysis demonstrates correspondence with vHIT-defined high-frequency vestibular dysfunction rather than independent diagnostic accuracy for AUVP. vHIT is not a standalone gold standard for AUVP, and AUVP remains a clinical diagnosis requiring integration of symptoms, bedside findings, auditory features, central signs, and objective vestibular testing. In particular, the reported sensitivity and specificity describe how closely caloric CP thresholds corresponded to the vHIT-based classification and should not be interpreted as diagnostic accuracy against an independent disease reference standard. Because caloric testing and vHIT assess vestibular function over fundamentally different frequency ranges (~0.003 Hz vs. 1–5 Hz), the two tests are not fully interchangeable, and a degree of discordance is expected and clinically informative rather than indicative of test error. We also did not perform a formal continuous correlation between caloric CP and vHIT gain asymmetry across the pooled cohort; because the two tests assess different frequency domains and vHIT gain exhibits a ceiling effect in the normal range, threshold-based comparison was considered more clinically interpretable than a linear correlation coefficient in this context.

Second, the comparison group consisted of symptomatic patients with acute dizziness/vertigo and normal vHIT findings rather than asymptomatic healthy controls. This design was appropriate for the clinical question addressed in this study, which concerned the correspondence between caloric and vHIT findings among patients presenting with acute vertigo. Nevertheless, the estimated specificity and discordant classification rates should be interpreted within this symptomatic clinical population.

Third, the present cohort included only patients evaluated within 72 h of symptom onset. This restriction reduced variability related to vestibular compensation and differential recovery of low- and high-frequency vestibular function, but it also limits generalizability to subacute or recovering AUVP. The proposed 30% CP threshold should therefore be applied primarily as an acute-phase interpretive benchmark and should not be generalized to later disease stages without further validation.

Finally, this was a retrospective single-center study including only patients who underwent both caloric testing and vHIT, which may have introduced selection bias. Patients older than 70 years were excluded to reduce confounding from age-related vestibular decline; however, this exclusion may limit generalizability to older populations. Prospective multicenter validation using standardized vHIT interpretation, independent clinical reference standards, and external cohorts will be valuable for confirming the broader applicability of these findings.

## 5 Conclusion

In this retrospective cohort of patients evaluated within 72 h of acute vertigo onset, a caloric CP threshold of approximately 30% showed the closest correspondence with vHIT-defined unilateral high-frequency vestibular dysfunction. This threshold reduced CP-abnormal/vHIT-normal discordant classifications among symptomatic vHIT-normal patients who did not receive a final diagnosis of definite peripheral or central vestibular disease, while preserving close correspondence with vHIT-defined unilateral vestibular dysfunction. These findings should not be interpreted as establishing an independent diagnostic CP threshold for AUVP, because vHIT was not used as an independent disease reference standard. Rather, the 30% CP threshold may serve as a practical interpretive benchmark for caloric CP in relation to vHIT-defined findings in the acute clinical setting. When caloric testing is used to provide the “unambiguous evidence” of a VOR deficit required by the Bárány Society criteria, a CP of approximately 30% represents a more appropriate practical benchmark than the conventional 25% for a result corresponding to vHIT-defined unilateral loss, although the partial correspondence between the tests indicates that they should be used complementarily rather than as freely interchangeable evidence. Prospective multicenter validation using independent clinical reference standards is warranted.

## Data Availability

The datasets supporting the conclusions of this article are available from the corresponding author upon reasonable request, subject to approval by the Institutional Review Board (IRB).
